# Who do you trust? Wild birds use social knowledge to avoid being deceived

**DOI:** 10.1126/sciadv.aba2862

**Published:** 2021-05-28

**Authors:** Filipe C. R. Cunha, Michael Griesser

**Affiliations:** 1 Department of Anthropology, University of Zurich, Zurich, Switzerland.; 2 Behavioural Ecology Group, Wageningen University & Research, Wageningen, Netherlands.; 3 State Key Laboratory of Biocontrol, Department of Ecology and School of Life Sciences, Sun Yat-sen University, Guangzhou, China.; 4 Animal Ecology, Department of Ecology and Genetics, Evolutionary Biology Centre, Uppsala University, Uppsala, Sweden.; 5 Department of Biology, University of Konstanz, Konstanz, Germany.; 6 Center for the Advanced Study of Collective Behavior, University of Konstanz, Konstanz, Germany.

## Abstract

Many species give deceptive warning calls, enabled by the high risk of ignoring them. In Siberian jays, a territorial, group-living bird, individuals give warning calls toward perched predators and mob them. However, intruding neighbors can emit these warning calls in the absence of predators to access food, but breeders often ignore these calls. Playback field experiments show that breeders flee sooner and return later after warning calls of former group members than those of neighbors or unknown individuals. Thus, breeders respond appropriately only to warning calls of previous cooperation partners. This mechanism facilitates the evolution and maintenance of communication vulnerable to deceptive signaling. This conclusion also applies to human language because of its cooperative nature and thus, its vulnerability to deception.

## INTRODUCTION

Many communication systems are vulnerable to deception, where signals are used to convey false information. In acoustic communication, deception frequently involves predator warning calls (*1*–*4*), made possible by the high cost of ignoring these calls (*1*). Deceptive calls are emitted out of context, for example, a warning call is emitted in the absence of a predator, allowing callers to gain access to resources, particularly food (*1*, *4*). Consequently, receivers pay a cost when responding to deceptive calls.

In communication systems where deception is potentially persistent and deceptive calls are used frequently, call recipients can learn to recognize deceptive calls. Recipients will stop responding to calls of unreliable individuals (*5*, *6*) or even punish them (*7*, *8*). However, establishing caller reliability through one’s own experience can be costly, and thus, receivers could rely on social information to infer caller reliability.

**Fig. 1:**
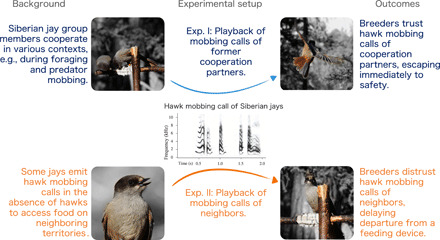
Natural history background, setup, and outcomes of experiments in Siberian jay. In this territorial, group-living species, individuals often try to access food on neighboring territories and do emit false predator warning calls (i.e., calls are usually given when mobbing perched hawks) in this context. Thus, breeders only trust warning calls of former group members (i.e., former cooperation partners). Photo credit: Filipe Cunha, Wageningen University & Research.

Here, we show that Siberian jay (*Perisoreus infaustus*) individuals use the social relationship with the caller to assess its reliability, thereby minimizing the risk of being deceived (Fig. 1). This bird species lives in stable family groups that, in addition to the dominant breeding pair, include up to five related and unrelated nonbreeders (*9*). Groups share a 0.5- to 1-km^2^ large all-purpose territory that members collectively defend against intruding neighbors (*9*, *10*). A particularly valuable resource on the territory is individually scatter-hoarded food (*11*), which individuals store in autumn to survive winter that lasts from October to early May, and breeders rely on to it successfully raise nestlings during the breeding season that lasts from March to May. Thus, the territory is indeed a critical resource for the survival of all group members (*12*), for the reproductive success of breeders (*9*), and for the future access to breeding positions for unrelated nonbreeders (*13*). Accordingly, the fitness of group members is intertwined or interdependent (*14*), and consequently, individuals should have a low incentive to deceive members of their own group.

Predation by hawks is the main driver of mortality in the study population (*12*). Upon detecting a perched hawk, individuals fly to a nearby tree, move up to its crown to approach, and mob the hawk for up to 5 min (*15*). Field experiments showed that Siberian jays have referential warning calls that are given specifically in response to perched hawks (*16*, *17*). However, individuals from neighbor groups utter perched hawk calls in the absence of predators to gain access to food (table S1). Thus, the information content of perched hawk calls may depend on caller identity.

We performed field experiments to compare the response of experienced breeders to playbacks of perched hawk calls of former group members versus unknown breeders (experiment I) and perched hawk calls of neighbors versus unknown breeders (experiment II) (Fig. 1). All these individuals were, at the time of the experiment, breeders. We used calls of former group members instead of current group members to have a more comparable intensity of social interactions between experimental and focal individuals. Group members almost constantly interact with each other (*9*, *10*) but only briefly once a day with neighbors (*10*) and never with unknown breeders. If social familiarity with the caller matters, then breeders will respond to calls of former group members and neighbors (*18*). If personal experience matters, then breeders will respond to calls of individuals that did not deceive them previously (*5*), including former group members and unknown breeders. Last, if caller credibility matters (i.e., recipients trust their calls), then breeders will only respond to calls of current or former group members.

## RESULTS

**Fig. 2:**
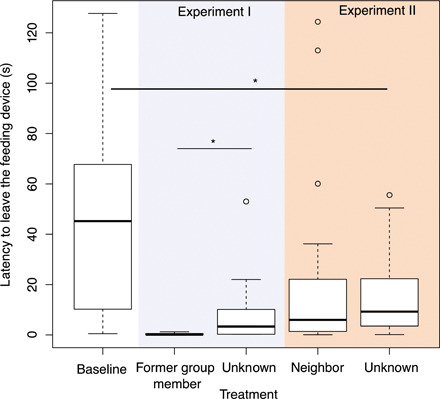
Latency of Siberian jay breeders to leave a feeding apparatus (in seconds) in the baseline setting, when exposed to a playback of a warning call of a former group member, a neighbor, and an unknown breeder. Lines in the boxes represent the median. Upper box limits indicate the third quartiles. The lower box limits indicate the first quartiles. The whiskers extend to 1.5 times the interquartile range. Circles designate outliers. Lines and asterisks indicate significant differences as shown in Table 2.

**Fig. 3:**
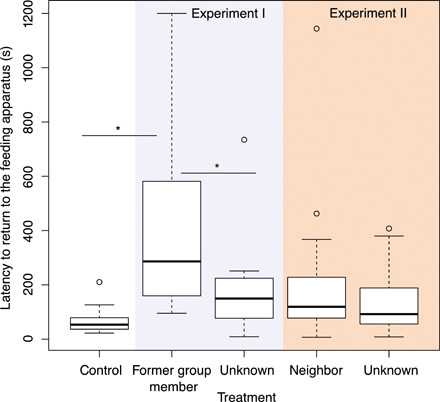
Latency of Siberian jay breeders to return to a feeding apparatus (in seconds) in the baseline setting, when exposed to a playback of a warning call of a former group member, a neighbor, and an unknown breeder. Lines in the boxes represent the median. Upper box limits indicate the third quartiles. The lower box limits indicate the first quartiles. The whiskers extend to 1.5 times the interquartile range. Circles designate outliers. We used a cutoff of 20 min for the latency to return. Lines and asterisks indicate significant differences as shown in Table 2.

The experimental setup mimicked a natural situation of jays foraging on an immobile animal carcass (see Fig. 1). We provided pig fat on a feeding apparatus and focused on breeders foraging in the absence of other group members. In this baseline setting, breeders foraged 45.1 ± 11.0 s (mean ± SE) on the feeding apparatus before flying off to scatter-hoard the collected food (Table 1 and Fig. 2) and returned after 68.0 ± 14.1 s (Table 1 and Fig. 3). When exposed to playbacks of warning calls given toward perched hawks, the social relationship to the caller determined the reaction of the receiver. Breeders that were exposed to warning calls of former group members had a shorter latency until leaving the feeding apparatus (0.3 ± 0.1 s) and took longer to return to it (437.2 ± 112.7 s) than when exposed to warning calls from unknown breeders (latency to leave: 9.2 ± 2.6 s; latency to return: 183.6 ± 55.5 s; Table 2 and Fig. 2 and Fig. 3). Breeders did not differ in their responses to warning calls of breeders from a neighboring territory and unknown breeders, both in their latency to leave the feeding apparatus (calls from neighbors: 20.1 ± 6.1 s; calls from unknown breeders: 15.2 ± 3.0 s; Table 2 and Fig. 2) and the latency to return to it (calls from neighbors: 193.0 ± 42.7 s; calls from unknown breeders: 131.7 ± 21.2 s; Table 2 and Fig. 3).

**Table 1:** Response of Siberian jay breeders to warning calls of former group members, neighboring breeders, and unknown breeders using the baseline setting as reference level. Linear mixed models (in the R package lme4) assessing the latency of breeders to leave and return to a feeding apparatus (in seconds, log transformed) after exposure to warning calls of former group members (exp. I), neighbors (exp. II), and unknown breeders (exp. I and II). degrees of freedom (df), subscripts. Receiver identity is included as a random factor.

	**Factor**	**Estimate**	**SE**	***t* value_(df)_**	***P* value**
Latency to leave the feeding apparatus					
	**Intercept**	**3.18**	**0.47**	**6.67_(87.93)_**	**<0.001**
Exp. I	**Baseline versus** **former group member**	**−4.83**	**0.67**	**−7.12_(82.58)_**	**<0.001**
	**Baseline versus** **unknown**	**−2.28**	**0.67**	**−3.37_(82.58)_**	**0.001**
Exp. II	**Baseline versus** **neighbor**	**−1.61**	**0.56**	**−2.87_(76.31)_**	**0.005**
	**Baseline versus** **unknown**	**−1.25**	**0.56**	**−2.23_(76.31)_**	**0.028**
	Random factor	Variance	SD		
	Receiver identity	0.28	0.53		
Latency to return to the feeding apparatus					
	**Intercept**	**4.03**	**0.30**	**13.80_(87.99)_**	**<0.001**
Exp. I	**Baseline versus** **former group member**	**1.73**	**0.44**	**3.89_(86.17)_**	**<0.001**
	Baseline versus unknown	0.62	0.44	1.39_(86.17)_	0.16
Exp. II	Baseline versus neighbor	0.64	0.37	1.74_(82.83)_	0.85
	Baseline versus unknown	0.41	0.37	1.11_(82.83)_	0.26
	Random factor	Variance	SD		
	Receiver identity	0.01	0.09		

**Table 2:** Response of Siberian jay breeders to warning calls of former group members and unknown breeders (exp. I) and of neighbors and unknown breeders (exp. II). Linear mixed models (in the R package lme4) assessing the latency (in seconds, log transformed) of breeders to leave and return to a feeding apparatus after exposure to warning calls. Receiver and caller identity are included as random factors.

**Factor**	**Estimate**	**SE**	***t* value_(df)_**	***P* value**
Latency to leave feeding apparatus (exp. I)				
**Intercept**	**−1.67**	**0.44**	**−3.75_(21.99)_**	**0.001**
**Former group member** **versus unknown**	**2.54**	**0.63**	**4.03_(20.60)_**	**<0.001**
Random factor	Variance	SD		
Receiver identity	<0.001	0.001		
Caller identity	0.007	0.279		
Latency to return to feeding apparatus (exp. I)				
**Intercept**	**5.75**	**0.31**	**18.05**_**(19.41)**_	**<0.001**
**Former group member** **versus unknown**	**−1.10**	**0.36**	**−3.08_(11.00)_**	**0.01**
Random factor	Variance	SD		
Receiver identity	0.44	0.66		
Caller identity	0.74	0.88		
Latency to leave feeding apparatus (exp. II)				
**Intercept**	**1.55**	**0.34**	**4.49_(46.87)_**	**<0.001**
Neighbor versus unknown	0.36	0.38	0.94_(27.00)_	0.36
Random factor	Variance	SD		
Receiver identity	1.30	1.14		
Caller identity	<0.001	<0.001		
Latency to return to feeding apparatus (exp. II)				
**Intercept**	**4.70**	**0.22**	**20.39_(46.34)_**	**<0.001**
Neighbor versus unknown	−0.21	0.30	−0.70_(40.77)_	0.48
Random factor	Variance	SD		
Receiver identity	<0.001	<0.001		
Caller identity	0.24	0.49		

Further analyses only assessing the responses to calls of former group members showed that neither the duration that individuals lived in the same group (*t* = 1.78, *P* = 0.10) nor kinship (differentiating family members from unrelated individuals; *t* = −0.41, *P* = 0.69) influenced the latency to leave the feeding apparatus. Similarly, the latency to return to the feeding apparatus was influenced by neither the duration that they lived in the same group (*t* = 0.96, *P* = 0.36) nor their kinship (*t* = −1.34, *P* = 0.21). Last, analyses only assessing the responses to calls of neighboring individuals showed that the time that they were neighbors influenced neither the latency to leave the feeding apparatus (*t* = −1.10, *P* = 0.28) nor the latency to return to it (*t* = 0.28, *P* = 0.78).

## DISCUSSION

Our results demonstrate that Siberian jays respond differently to playbacks of warning calls depending on the social relationship to the caller. Breeders immediately escape to safety when exposed to warning calls from former group members but not when exposed to warning calls from neighbors or unknown breeders. Siberian jays are familiar with all their neighbors and encounter them on a daily basis (*10*), but neighbors are more likely to give deceptive warning calls than individuals from their own group (table S1). Moreover, neighbors compete for space and the associated resources (*10*, *19*). Thus, familiarity alone does not breed trust, but Siberian jays trust only warning calls of former cooperation partners.

Breeders immediately responded to warning calls of former group members that they did not interact with for 2 to 5 years. Similarly to Siberian jays, other species can have a long-term memory of social information (*20*–*22*). Our results suggest that this social memory allows individuals to make informed decisions in novel situations and, thus, to avoid being deceived. Clearly, non–group members also give perched hawk calls in response to life hawks, and immediately seeking safety would be beneficial. The cost of ignoring their warning calls is therefore not negligible. However, the rate of deceptive calls (observed about every fifth day) is much higher than the rate of encountering a perched hawk (observed about once every 8.5 months) (*12*). It is therefore very unlikely that perched hawk calls given by neighbors would designate a life hawk.

The absence of response to warning calls of neighbors raises the question of why false warning calls are given at all. A previous playback experiment exposed breeders foraging together with juveniles to perched hawk calls of unknown breeders (*17*). All breeders immediately left the feeding apparatus (*17*). This response may reflect that juvenile Siberian jays learn to recognize predators by observing group members interacting with life predators (*19*, *23*). Thus, juveniles also probably have to learn to respond to perched hawk calls, and therefore, breeders accompanied by juveniles respond to all perched hawk call independent of caller identity. Our experiment does not allow us to assess whether trust is contingent on experience or whether this can be learned from others.

The judgment of caller trustworthiness could be based on three proximate mechanisms. Individuals could assess the trustworthiness of callers on the basis of acoustic similarity with the own call (i.e., a group signature), by remembering individual calls of former group members, or a general categorization of caller trustworthiness. In other species, vocal group signatures have been shown to facilitate individual recognition and increase social cohesion during conflicts with neighboring groups (*24*, *25*). Post hoc analyses show that mobbing calls of individuals sound different (table S2 and fig. S2) and that mobbing calls of former group members are acoustically more similar than those of neighbors or unknown individuals (table S3). This finding indicates that perched hawk calls are learned from others, and if so the case, juveniles learn perched hawk calls particularly from male breeders in their group, as they give most mobbing calls (*15*). Thus, call similarity is a proximate expression of having lived together, also reflecting the associated interdependence. However, this mechanism would prevent the development of proper group signature in Siberian jays. Male and female breeders are likely to call differently, as they usually grow up on different territories (98.7% of breeder pairs in our population during the time of this study). Nevertheless, our experiment does not allow us to distinguish among these potential proximate mechanisms.

Trusting only signals of cooperation partners may facilitate the evolution and maintenance of communication systems vulnerable to deceptive signaling. A prime example of such a communication system is human language, due to its cooperative, prosocial nature (*26*). A basic function of human language is declarative communication [i.e., individuals share facts or knowledge that can be true of false (*27*)], and thus, language can only be maintained if deception is minimized, else it collapses. In the case of Siberian jays, knowledgeable individuals accompanied by juveniles always respond to perched hawk calls (*17*), facilitating the learning of call meaning. Clearly, this setting is enabling deception, but only trusting warning calls of cooperation partner limits the opportunities for deception. Similarly to Siberian jays, humans also are more likely to trust individuals that belong to the same group and therefore are more likely to be cooperation partners (*28*, *29*). Thus, vulnerability for deception may also be a driver of rapid diversification of languages and facilitate the formation of dialects, being signifiers for local groups of cooperators.

## METHODS

This study was carried out in an individually color-banded population of Siberian jay near Arvidsjaur, Northern Sweden (65°40′N, 19°0′E). Birds in this population have been followed since 1989, and here, we used data collected between August and October, years 2014, 2015, and 2017, in a total of 35 groups. Predation is the main cause of mortality in our study population, and most individuals are killed by accipiter hawks (*12*). Upon encountering a hawk, Siberian jays give referential warning calls that are specific to the behavior of the hawk, allowing call recipients to respond appropriately (*17*).

### Recording perched hawk calls

We recorded the vocal response of 20 male and 20 female breeders to a taxidermized perched sparrowhawk (*Accipiter nisus*) model while foraging alone on a feeding apparatus (see Fig. 1). We placed the feeding apparatus 3 to 4 m away from the predator model (covered with a camouflaged cloth) on a 1.5-m-high pole and 2 m away from the nearest tree. We fed other group members large pieces of food, which subsequently flew off to process and cash the food. When all other group members were out of sight, we uncovered the sparrowhawk model when the focal individual was approaching the feeding apparatus. We recorded its vocalizations for a maximum of 3 min or until it left the experimental area using a Sony PCM-10 recorder connected to a directional Telinga Pro microphone with a 58-cm-diameter parabolic dish.

### Playback experiments

We created playback sequences of 93-s length from the recorded warning calls using Adobe Audition software. The calls were arranged in the same order that they were recorded, adding 4 s of silence between each call (i.e., the mean spacing of different calls given in natural mobbing sequences) and 3 s of silence in the beginning of each track.

To assess the influence of caller identity on the response of breeders, we placed a loudspeaker with Bluetooth connection (Philips SB5200) 3 to 4 m away from the feeding apparatus on the ground. We played the mobbing call sequences stored on an iPod (Apple, Cupertino, CA) when the focal individual was alone on the feeding apparatus and other group members were out of sight. The playback volume was set so that the sequences were, at maximum, heard at a distance of 50 m from the speaker, and the volume was kept at the same level across all trials. This volume corresponds to natural mobbing calls that are audible at a distance of ca. 50 m. All experimental trials were recorded with a video camera. On the basis of these video recordings, we measured the latency to respond, i.e., the time in seconds that the focal individual took to leave the feeding apparatus after the start of the playback, and the latency to return, i.e., the time in seconds that the focal individual took to return to the feeding apparatus, using the software ELAN (*30*). We used a cutoff of 20 min for the latency to return.

We carried out two experiments to assess the effect of caller identity. We exposed breeders to call sequences of former group members and unknown breeders (experiment I, conducted autumn 2014) or to call sequences of neighbors and unknown breeders (experiment II, conducted autumn 2015). The call used in the “unknown breeder” treatment was recorded from an individual of a group that was at least 6 km away from the recipient and therefore well beyond the area where individuals may interact (*10*). We used warning calls of *N* = 33 individuals for the unknown treatment and use them in *N* = 40 trials.

In experiment I, we chose *N* = 6 pairs of individuals that lived in the same group 2 to 5 years before the experiment. These individuals were breeders at the time of the experiment and never lived on adjacent territories before the experiment. We exposed each of the 12 individuals to a playback sequence from a former group member and an unknown breeder at least 2 days apart. The order of the playback treatments was counterbalanced. For experiment II, we selected 28 pairs of breeders that had never lived in the same group but were breeders in adjacent territories at the time of the experiment. Neighboring groups encounter each other about once a day (*10*), and thus, breeders are familiar with all individuals from neighboring groups. We used the same setup as for experiment I but exposed focal individuals to playbacks of warning calls from a neighbor and an unknown breeder.

We recorded the natural foraging behavior of individuals at the feeding apparatus (i.e., baseline setting) using the same setup as in both experiments in autumn 2017. We isolated one of the breeders in the focal group. Then, we assessed the time that an individual spent on the feeding apparatus before leaving it while all other group members were absent and the time it took to return to the feeding apparatus.

### Statistical analyses

All analyses and plots were done in the statistical software R version 3.5.2 (*31*). We used linear mixed models in the package “lme4” (*32*) to test the latency to respond and the latency to return in response to warning calls of a former group member, a neighbor, and an unknown breeder and in the baseline setting. We used the package “lmerTest” to assess the degrees of freedom from the models (*33*). We transformed the latency to react and the latency to return into a logarithmic scale using the “log” function to fulfill the model assumptions. Collinearity effects assessed through VIFs (Variance Inflation Factor) were negligible (tested with package “car”) (*34*). We included receiver identity in all models as a random factor. For the models that separately assessed the response in experiments I and II, caller identity was included as a random factor to control for the repeated use of *N* = 7 mobbing call sequences. During the baseline setting, we did not playback warning calls, and thus, caller identity was not included in these analyses.

We first analyzed whether the latency to react to warning calls in experiments I and II was different from the time that individuals spent on the feeding apparatus in the baseline setting, i.e., without being exposed to warning calls. Then, we analyzed whether the latency to return to the feeding apparatus after being exposed to warning calls in experiments I and II was different from the baseline setting.

To assess the differences in the latency to respond and the latency to return in both experiments, we separately analyzed each experiment. For experiment I, we analyzed the difference in the latency (i) to leave the feeding apparatus and (ii) to return to the feeding apparatus after being exposed to a warning call of a former group member and an unknown breeder. For experiment II, we analyzed the difference in the latency (i) to leave the feeding apparatus and (ii) to return to the feeding apparatus after being exposed to a warning call of a neighboring breeder and an unknown breeder.

For experiment I, we also tested whether the period of time that individuals have been neighbors (in months) influenced their latency to leave and to return to the feeding apparatus. We used a linear model using the function “lm” and included only individuals exposed to calls from neighbors. For experiment II, we tested whether the latency to leave and to return to the feeding apparatus were influenced by the period of time that they had lived together in the same group (in months) and their kinship [family members (parent-offspring and sibling-sibling) versus unrelated individuals]. We used linear models using the function lm and included only the experimental group exposed to a warning call of a former group member.

All experiments were recorded with video cameras so that the assessment of the latency to respond and return to the feeder could be precisely measured by selecting the frame where the focal individual started to take off from the feeder and respectively landed on the feeder. We conducted an observer reliability test in a sample of the trials (*n* = 13) by calculating the intraclass correlation coefficients (ICCs) between F.C. and an unbiased observer unfamiliar with the study using the “irr” package (*35*). The ICCs ranged between 0.98 (latency to respond) and 1.00 (latency to return), showing that our protocol allowed unbiased and repeatable collection of data.

## SUPPLEMENTARY MATERIALS

Supplementary material for this article is available at http://advances.sciencemag.org/cgi/content/full/7/22/eaba2862/DC1
